# Case Report: Ofatumumab treatment for concomitant multiple sclerosis and idiopathic thrombocytopenic purpura

**DOI:** 10.3389/fimmu.2025.1515776

**Published:** 2025-03-11

**Authors:** Yuki Yokota, Makoto Hara, Hideto Nakajima

**Affiliations:** Division of Neurology, Department of Medicine, Nihon University School of Medicine, Tokyo, Japan

**Keywords:** anti-CD20 monoclonal antibodies, multiple sclerosis, thrombocytopenia, idiopathic thrombocytopenic purpura, ofatumumab

## Abstract

**Background:**

Herein, we detail our experience with a unique patient with concomitant multiple sclerosis (MS) and idiopathic thrombocytopenic purpura (ITP) treated with ofatumumab, which resulted in stable disease activity and platelet count normalization.

**Case presentation:**

A 21-year-old Japanese woman presented with medial longitudinal fasciculus syndrome and was subsequently diagnosed with MS. She was treated with methylprednisolone pulse therapy (1,000 mg/day for 5 days). During her first hospitalization, her platelet count was low (40 × 10^9^/L). Based on investigations, serologic findings, and bone marrow aspiration, she was diagnosed with ITP. Following methylprednisolone treatment, oral prednisolone was initiated and gradually tapered. Glatiramer acetate was used as a disease-modifying drug (DMD). As prednisolone was tapered off, the platelet count decreased correspondingly. The clinical course included two MS relapses, each of which was treated with a methylprednisolone pulse and DMD adjustments (the DMT was sequentially switched from glatiramer acetate to dimethyl fumarate, then fingolimod, and finally natalizumab). Despite an initial recovery of the platelet count following these interventions, the platelet count declined correspondingly with the prednisolone dose reduction. Finally, the DMD was switched to ofatumumab, an anti-CD20 monoclonal antibody with pharmacological similarities to rituximab, a second-line treatment for ITP. After the initiation of ofatumumab, the patient remained clinically stable with no further MS relapses, and her platelet count stabilized over 2 years.

**Conclusions:**

Herein, we report our experience with a novel case of MS concomitant with ITP that was safely treated with ofatumumab. Considering the pharmacological similarities of ofatumumab to rituximab (a second-line treatment for ITP), anti-CD20 monoclonal antibodies such as ofatumumab could be a promising treatment option for cases of MS concomitant with ITP.

## 1 Introduction

Multiple sclerosis (MS) is a chronic, immune-mediated disease that affects the central nervous system, often leading to neurological disabilities in young adults. Moreover, comorbid autoimmune diseases such as thyroiditis, rheumatoid arthritis, inflammatory bowel disease, and autoimmune diabetes have been documented in patients with MS (1). Although reports on the coexistence of MS and idiopathic thrombocytopenic purpura (ITP) are limited (2), one study indicates a significantly higher prevalence of MS in patients with ITP than in the general population (1.4 per 100 patients with ITP versus an expected population prevalence of 0.058 to 0.077) (3). However, there are no established treatment strategies for MS patients with concomitant ITP, making their management particularly challenging. Additionally, there are reports of drug-induced thrombocytopenia in patients with MS (4–8), although the underlying mechanism has yet to be elucidated. One potential mechanism of alemtuzumab-induced thrombocytopenia is the imbalance in immune reconstitution after lymphocyte depletion. Alemtuzumab induces rapid and profound depletion of B and T cells, a hallmark of its therapeutic effect. During the recovery phase, B cells repopulate more quickly than T cells, particularly regulatory T cells, leading to an environment of inadequate T cell-mediated control. This accelerated and disproportionate recovery of B cells leads to the proliferation of autoreactive B cells, producing pathogenic antibodies. Such a process could contribute to the development of secondary autoimmune conditions, including thrombocytopenia (9). Furthermore, there are no established treatment strategies for these secondary autoimmune conditions.

Herein, we present the case of a patient with relapsing-remitting MS (RRMS) who had concomitant ITP and was successfully treated with ofatumumab. The highly active RRMS of the patient remained stable, and her thrombocytopenia also improved with ofatumumab treatment. Considering the pharmacological similarities of ofatumumab to rituximab, a second-line treatment for ITP (10), anti-CD20 monoclonal antibodies such as ofatumumab may represent a promising treatment option for cases of MS concomitant with ITP.

## 2 Case presentation

**Figure 1:**
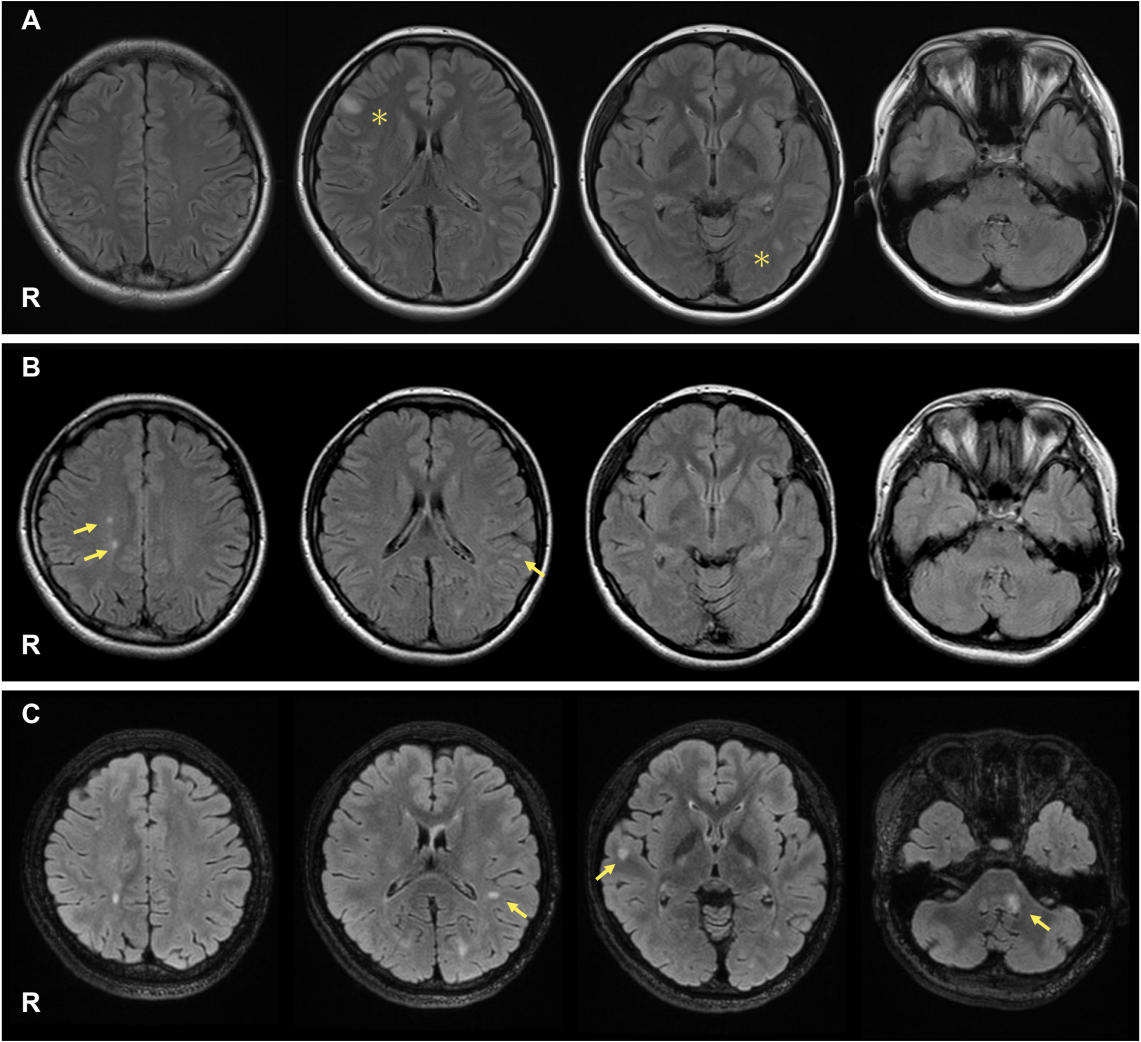
On admission, her brain MRI revealed high-intensity lesions in the right frontal cortex and around the left lateral ventricle (**(A)**, asterisks). During the first MS relapse, new T2 lesions appeared in the right parietal and left temporal lobes (**(B)**, arrows). During the second MS relapse, new T2 lesions were observed around the left lateral ventricle, right temporal lobe, and left middle cerebellar peduncle (**(C)**, arrows).

A 21-year-old Japanese woman presented to our hospital with diplopia. Two years earlier, she experienced dysesthesia in her left leg, a first episode of neurologic symptoms that might be related to MS. At that time, brain magnetic resonance imaging (MRI) revealed unremarkable findings, and the symptoms resolved spontaneously. Her neurological examination revealed limited adduction of the left eye and diplopia on her right gaze, which are both consistent with left medial longitudinal fasciculus syndrome. Her Expanded Disability Status Scale (EDSS) score was 2.0. There were no other neurological findings, including dysesthesia, muscle weakness, or ataxia. Cerebrospinal fluid (CSF) analyses revealed 14 white blood cells/mm^3^, 35 mg/dL total protein, and 104 pg/mL of myelin basic protein. The IgG index was 1.65 (cutoff: <0.67), and oligoclonal IgG bands in the CSF were positive. Her brain MRI revealed high-intensity lesions in the right frontal cortex and around the left lateral ventricle (**Figure 1A**). Her spinal MRI revealed no evidence of demyelinating lesions in the spinal cord. Laboratory tests revealed a white blood cell count of 4.5 × 10^9^/L, a hemoglobin (Hb) level of 9.7 g/dL, and a platelet count of 40 × 10^9^/L. Liver, kidney, and thyroid function tests were all normal, and her glycosylated hemoglobin (HbA1c) level was 5.5%. Immunological tests were negative for specific antibodies, including the antinuclear antibody, anti-dsDNA antibody, anti-SS-A antibody, anti-cardiolipin/β2-glycoprotein I complex antibody, MPO-ANCA, and PR3-ANCA. Antibodies against aquaporin-4 and myelin oligodendrocyte glycoprotein were not detected in either serum or CSF using a cell-based assay with live HEK293 cells. Epstein-Barr (EB) virus anti-VCA IgM was 0.3 (–), anti-VCA IgG was 4.6 (+), and anti-EBNA IgG was 3.3 (+), indicating a past infection. She was diagnosed with RRMS and received methylprednisolone pulse therapy (1,000 mg/day for 5 days). On Day 1 (symptom onset), the platelet count was 40 × 10^9^/L. Following methylprednisolone pulse therapy, it increased to 278 × 10^9^/L on Day 9. Her symptoms gradually improved, and she was discharged two weeks after the onset of symptoms. Subsequently, treatment with glatiramer acetate (20 mg/day) was initiated; however, a month later, she was readmitted due to an MS relapse, manifesting as new neurological deficits, including dysesthesia and decreased tactile sensation in her left lower extremity (EDSS 2.0). Her brain MRI revealed new high-intensity lesions in the right parietal and left temporal lobes of her brain (**Figure 1B**). Therefore, methylprednisolone pulse therapy was readministered, and the DMD was switched to dimethyl fumarate (initiated at 240 mg/day and increased to a dose of 480 mg/day after one week). However, due to her impaired liver function (AST 100 U/L, ALT 159 U/L), it was switched to fingolimod (0.5 mg/day).

During this period, her platelet count dropped below 50 × 10^9^/L on Day 99, which was designated as Day 1 of the first relapse. Serologic findings revealed no evidence of organ failure, hematologic malignancy, rheumatic disease, or infectious diseases such as hepatitis B and C, HIV, or acute cytomegalovirus infection. She tested positive for platelet-associated IgG autoantibodies and negative for *Helicobacter pylori* IgG antibody. Bone marrow aspiration revealed normal cellularity, no atypical cells, prominent megakaryocytes, and normal cytogenetics. Based on the laboratory findings, other autoimmune diseases such as systemic lupus erythematosus were ruled out due to the absence of clinical features and negative serologic tests. Drug-induced thrombocytopenia was considered unlikely due to the lack of evidence of causative drugs in her medical history. In addition, infections that could cause thrombocytopenia were excluded based on negative serologic findings. Finally, she was diagnosed with ITP. Her platelet counts improved with corticosteroid treatment, increasing to 124 × 10^9^/L on Day 5 following methylprednisolone pulse therapy, but decreased again to 35 × 10^9^/L on Day 77 when the dosage was reduced.

Approximately one year after initiating fingolimod, she experienced a second MS relapse. Her neurological examination revealed limited abduction of her left eye, decreased tactile sensation on her left hemiface, left facial muscle weakness, and reduced audition in the left ear (EDSS 3.0). Brain MRI revealed new high-intensity lesions around the left lateral ventricle, right temporal lobe, and left middle cerebellar peduncle. Some lesions showed contrast enhancement with T1-weighted gadolinium (Gd) (**Figure 1C**; Gd-enhancement is not shown).

Therefore, we administered methylprednisolone pulse therapy followed by oral prednisolone. We considered transitioning from fingolimod to alternative therapies such as ofatumumab, ocrelizumab, or rituximab; however, they were not available in Japan at that time. Thus, we alternatively switched to natalizumab (300 mg every 4 weeks) after confirming the absence of detectable antibodies against the JC virus, as indicated by an anti-JC virus antibody index of 0.16. Subsequently, there were no MS relapses, and the clinical course of MS stabilized, with low platelet counts (between 60 × 10^9^/L and 100 × 10^9^/L). Given the mild severity of her thrombocytopenia, maintenance immunotherapy with corticosteroids was considered unnecessary for ITP.

**Figure 2:**
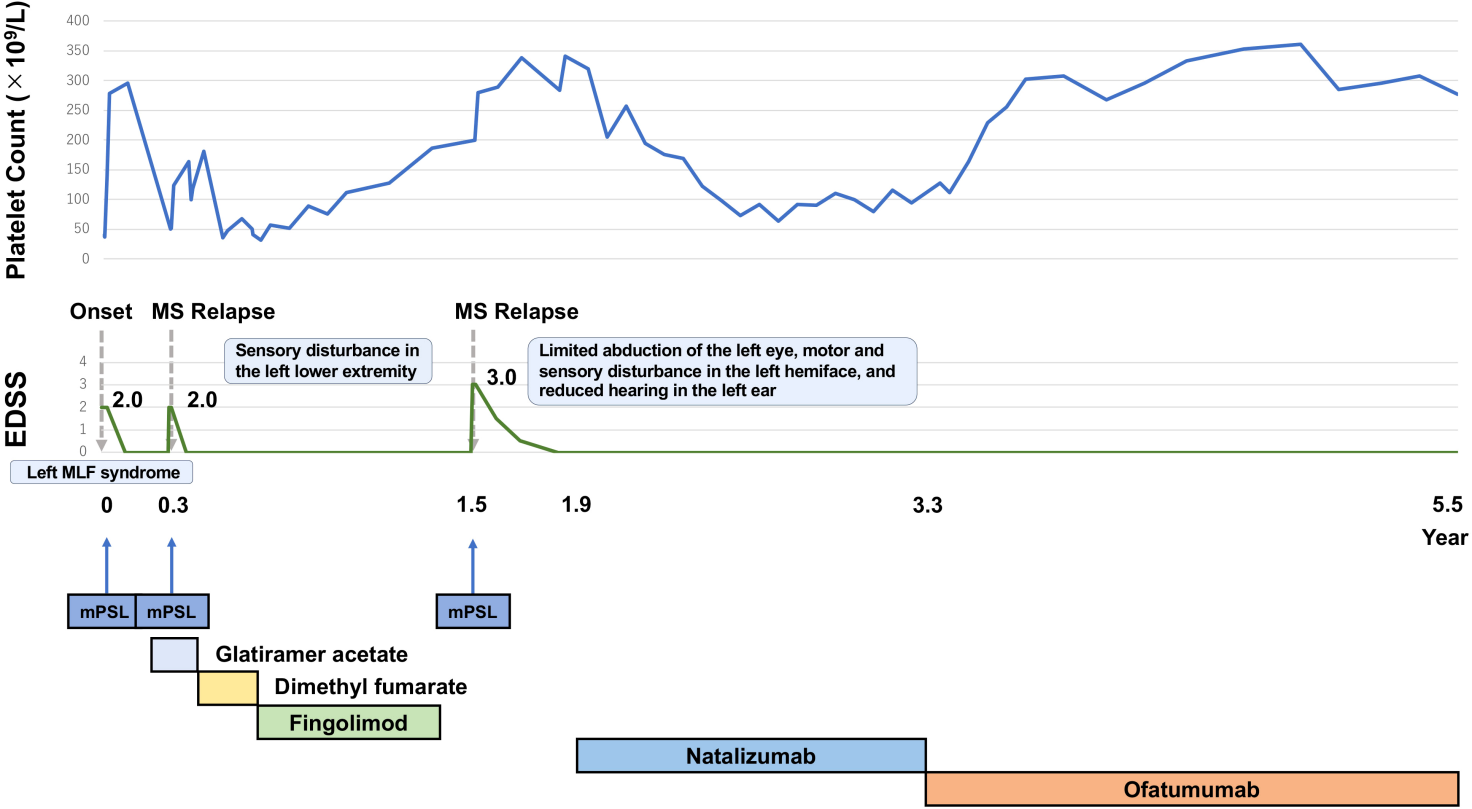
The patient experienced three clinical events, each of which was treated with methylprednisolone pulse therapy, leading to platelet count increments after each treatment course. Due to high MS activity, the DMD was switched to natalizumab. After switching to natalizumab, MS activity remained stable. Platelet counts increased to normal levels and have remained normal since the end of ofatumumab induction. MS activity has remained stable, comparable to the period on natalizumab.

Approximately one year later, ofatumumab became available in Japan, whereas ocrelizumab and rituximab remained unavailable. Considering its pharmacological similarities to rituximab (a second-line treatment for ITP), we switched from natalizumab to ofatumumab (20 mg every 4 weeks) with a shared decision-making process. At the time of the switch, the anti-JC virus antibody index was 0.22, and detectable antibodies remained negative. The decision to switch was based on the potential benefits of ofatumumab in MS and ITP, and the patient’s mild, but consistent thrombocytopenia, which remained unchanged during natalizumab treatment (**Figure 2**). In addition, long-term use of natalizumab requires careful consideration due to its associated risk of progressive multifocal leukoencephalopathy, prompting a switch to ofatumumab, which is classified as a high-efficacy therapy. Since then, MS activity has remained stable, similar to the period on natalizumab, with no MS relapses to date. Her platelet count has remained normal over 2.2 years of follow-up since completing ofatumumab induction (**Figure 2**).

## 3 Discussion

Herein, we describe a case of concomitant MS and ITP that was successfully treated with ofatumumab, a monoclonal anti-CD20 antibody. After the initiation of ofatumumab, the patient’s disease activity remained stable, with no MRI progression, no clinical relapses of MS, and no worsening of the EDSS score, achieving no evidence of disease activity-3 (NEDA-3). Additionally, the ITP-induced thrombocytopenia also improved.

Patients with ITP have a significantly higher prevalence of MS compared to the general population. Several studies have identified an association between platelet function and MS. For instance, platelet activation has been reported in the peripheral blood of MS patients (11), and platelet-related components have been detected within or surrounding MS lesions (12). However, the precise relationship between these findings and the pathophysiology of MS remains unclear. There is a paucity of reports on patients with concomitant MS and ITP. We reviewed previous cases of MS occurring alongside ITP (**Table 1**), and performed a literature search using PubMed with the terms “multiple sclerosis” AND “idiopathic thrombocytopenic purpura” AND “case report” to identify relevant cases. We also searched for the terms “ofatumumab” AND “ITP” to see if similar cases had been reported. Sahraian et al. reported the cases of three patients with MS, including one with pediatric-onset disease, who also had chronic ITP. Two of these patients had RRMS, and the other had secondary progressive MS. One patient had severe MS with an EDSS score of 7.5, while the other two had a favorable clinical course (EDSS 0-1) (2). Cochran et al. also reported the case of a 21-year-old female patient with severe chronic ITP concomitant with RRMS. Their focus was on the treatment protocol for ITP using ofatumumab desensitization, without including details on the clinical course of concomitant MS. Additionally, the outcomes of MS were not discussed in these reports (13). In contrast, we have provided a detailed account of how our patient achieved favorable outcomes, namely NEDA-3, over a two-year period, after being treated with ofatumumab for highly active RRMS and ITP.

**Table 1:** Literature review of MS cases concomitant with ITP.

	Sahraian et al. (2)			Cochran et al. (13)	Present case
Age/Sex	26/F	36/F	33/F	39/F	26/F
Age of MS onset	16	23	31	ND	21
MS onset presentation	INO	Walking difficulties	Tongue paresthesia	ND	Left medial longitudinal fasciculus syndrome
MS type	RR	SP	RR	ND	RR
Age of ITP diagnosis (years)	6	36	27	39	21
Treatment regimen for MS at ITP onset	ND	No treatment	ND	ND	No treatment
ITP type	Chronic	Chronic	Chronic	Chronic	Chronic
Treatment	Azathioprine	Oral prednisolone	IFN β-1a	Ofatumumab	Ofatumumab
EDSS	1	7.5	0	ND	0
Follow-up period (months)	ND	ND	ND	ND	70

EDSS, Expanded Disability Status Scale; IFN, interferon; INO, internuclear ophthalmoplegia; ITP, idiopathic thrombocytopenic purpura; MS, multiple sclerosis; ND, not described; RR, relapsing-remitting; SP, secondary progressive.

It should also be noted that drug-induced thrombocytopenia has been reported with the use of DMDs for MS, including interferon-β-1a (2, 14), natalizumab (4), glatiramer acetate (5), fingolimod (6, 8), and alemtuzumab (7). Thrombocytopenia associated with MS can occur as a complication of ITP or be an adverse event of these drugs. In our case, thrombocytopenia was revealed at MS onset before DMD administration, and concomitant ITP was eventually confirmed with the positive results of anti-platelet IgG. Additionally, ofatumumab-induced thrombocytopenia was not observed over two years of follow-up. No confirmed cases of ofatumumab-induced thrombocytopenia in MS have been reported in phase 3 trials (15) and post-marketing surveillance. Ofatumumab is a fully human antibody targeting B cells, which have antibody-dependent cellular cytotoxicity and complement-dependent cytotoxicity (16). Immunogenicity can lead to the production of anti-drug antibodies (ADAs), which may interfere with a drug’s efficacy or induce adverse effects. Ofatumumab (a fully human monoclonal antibody) falls into the least immunogenic category of monoclonal antibodies. Cotchett et al. also suggested that the minimal immunogenicity of ofatumumab may lead to a lower incidence of ADA production compared with the other not fully human monoclonal antibodies such as rituximab or ocrelizumab (16), which might be one of the reasons why no confirmed cases of ofatumumab-induced thrombocytopenia have been reported in patients with MS. In contrast, instances of rituximab-induced thrombocytopenia have been documented in the literature (17–21).

As for the treatment of MS and ITP, splenectomy and rituximab are known therapeutic options for ITP, especially in refractory cases (10). However, physicians should be aware that Matsui et al. reported the case of a patient who developed MS after splenectomy for ITP (22). The supposed mechanism is that splenectomy reduces the number of helper T-cells and causes a decrease in helper T-cell activity, possibly leading to the development of autoimmune phenomena (23, 24). Therefore, splenectomy should be avoided in patients with ITP concomitant with MS in favor of other treatment modalities. On the other hand, rituximab reduces platelet-associated antibody titers (25) and improves abnormalities in T-cell subsets in patients with ITP (26). From a mechanistic perspective, other anti-CD20 monoclonal antibodies may also be effective, but rituximab remains the only approved second-line treatment for ITP. Ofatumumab, another anti-CD20 monoclonal antibody, is also effective in MS by modulating the response and migratory potential of inflammatory T-cells (27). Considering the therapeutic similarities between ofatumumab and rituximab, particularly the efficacy in modulating T-cell abnormalities, ofatumumab could be a promising treatment option for stabilizing the activity of MS and ITP. In addition, ocrelizumab has emerged as another viable treatment option. While ocrelizumab is a humanized monoclonal antibody, ofatumumab, as a fully human monoclonal antibody, may offer advantages in terms of immunogenicity, potentially reducing the risk of anti-drug antibody production. This distinction may provide a better safety profile for ofatumumab, particularly regarding the risk of drug-induced thrombocytopenia.

We did not test for platelet-associated IgG autoantibodies after initiation of ofatumumab treatment. As a result, we could not confirm its effect on one of the underlying mechanisms of ITP. We acknowledge this as a limitation of our case.

In conclusion, anti-CD20 monoclonal antibodies such as ofatumumab may safely offer an effective treatment option for atypical cases of MS with concomitant ITP.

## Data Availability

The datasets presented in this article are not readily available because of ethical and privacy restrictions. Requests to access the datasets should be directed to the corresponding author/s.
